# Draft Whole-Genome Sequence of “*Candidatus* Liberibacter asiaticus” Strain TX2351 Isolated from Asian Citrus Psyllids in Texas, USA

**DOI:** 10.1128/genomeA.00170-17

**Published:** 2017-04-13

**Authors:** Madhurababu Kunta, Zheng Zheng, Fengnian Wu, John V. da Graca, Jong-Won Park, Xiaoling Deng, Jianchi Chen

**Affiliations:** a Texas A&M University–Kingsville Citrus Center, Weslaco, Texas, USA; b South China Agricultural University, Guangzhou, China; c USDA, ARS, San Joaquín Valley Agricultural Sciences Center, Parlier, California, USA

## Abstract

We report here the draft genome sequence of “*Candidatus* Liberibacter asiaticus” strain TX2351, collected from Asian citrus psyllids in south Texas, USA. The TX2351 genome has a size of 1,252,043 bp, a G+C content of 36.5%, 1,184 predicted open reading frames, and 52 RNA genes.

## GENOME ANNOUNCEMENT

Huanglongbing (HLB), or citrus greening disease, is a serious threat to citriculture worldwide (1–3). In the United States, HLB has been associated with a phloem-inhabiting alphaproteobacterium, “*Candidatus* Liberibacter asiaticus,” vectored by Asian citrus psyllid (ACP) (*Diaphorina citri* Kuwayama). In the Americas, “*Ca.* Liberibacter asiaticus” was first discovered in Brazil in 2004 (4) and later in Florida in 2005 (5) and in California in 2012 (6). HLB was first detected in a Valencia sweet orange tree in a commercial orchard in San Juan, Texas, USA, in 2012 (7, 8), and it has now spread to all citrus-growing regions in the Rio Grande Valley of south Texas. Because of its unculturable nature, the characterization of “*Ca.* Liberibacter asiaticus” relies heavily on genome sequence analyses. Whole-genome sequences of “*Ca.* Liberibacter asiaticus” have been reported from strains found in Florida (9, 10) and California (11, 12). Here, we report a draft whole-genome sequence of “*Ca.* Liberibacter asiaticus” strain TX2351, isolated directly from ACP in south Texas.

ACPs carrying “*Ca.* Liberibacter asiaticus” strain TX2351 were originally collected in May 2016, by USDA-APHIS-PPQ Citrus Health Response Program inspectors on a Mexican lime tree (*Citrus aurantiifolia*) showing typical HLB symptoms in McAllen, Texas. Total DNA from five field-collected ACPs was extracted using the BioSprint 96 DNA blood kit (Qiagen, Germantown, MD, USA) and tested positive for “*Ca.* Liberibacter asiaticus” (cycle threshold = 23.7) by the PCR method of Li et al. (13). A quantity of DNA was enlarged using Illustra GenomiPhi version 2 DNA amplification kits (GE Healthcare, Waukesha, WI, USA). Whole-genome sequencing was performed on an Illumina MiSeq platform (Illumina, Inc., San Diego, CA, USA). The MiSeq sequencing yielded a total of 4.27 × 10^7^ reads with an average size of 251 bp. With the help of the whole-genome sequences of “*Ca.* Liberibacter asiaticus” strains Psy62 (9) and A4 (14) and 2 “*Ca.* Liberibacter asiaticus” phages/prophages (SC1 and SC2) (15), a total of 32,768 reads were identified and collected using Bowtie2 version 2.2.9 (16). Sequence assembly was performed using a combination of both Velvet 1.2.0 (17) and CLC Genomics Workbench version 7.5 software. The assembly contained 71 contigs, ranging from 1,139 bp to 121,132 bp in size with ~6.90×, comprising a total of 1,252,043 bp with a G+C content of 36.5%. Annotation was performed using the RAST server (http://rast.nmpdr.org) (18), which predicted 1,184 open reading frames and 52 RNA genes.

### Accession number(s).

This whole-genome shotgun project has been deposited at DDBJ/EMBL/GenBank under the accession number MTIM00000000. The version described here is the first version, MTIM01000000.
